# Clinical characteristics and courses of 200 patients hospitalized for COVID-19 during the second and third waves at Fujita Health University Okazaki Medical Center in Japan

**DOI:** 10.20407/fmj.2021-018

**Published:** 2022-05-25

**Authors:** Masamichi Hayashi, Sayako Morikawa, Yusuke Goto, Takazumi Yoshida, Yutaro Kimura, Taku Kawabe, Seiichiro Tsuzuki, Kazuyoshi Imaizumi

**Affiliations:** 1 Department of Internal Medicine, Division of Respiratory Medicine, Fujita Health University, School of Medicine, Okazaki, Aichi, Japan; 2 Department of Respiratory Medicine I, Fujita Health University, School of Medicine, Toyoake, Aichi, Japan; 3 Department of Respiratory Medicine II, Fujita Health University, School of Medicine, Nagoya, Aichi, Japan; 4 Department of General Practice, Fujita Health University, School of Medicine, Okazaki, Aichi, Japan; 5 Department of General Internal Medicine and Emergency, Fujita Health University, School of Medicine, Okazaki, Aichi, Japan

**Keywords:** COVID-19, Second wave, Third wave, Dexamethasone, Remdesivir

## Abstract

**Objectives:**

There are few reports about patients hospitalized for COVID-19 in Japan. We investigated 200 patients hospitalized for COVID-19 over a 6-month period with the aim of elucidating their clinical characteristics and clinical courses.

**Methods:**

The study cohort comprised 200 patients hospitalized for COVID-19 during a 6-month period. We examined baseline characteristics, source of transmission, preadmission course, initial symptoms, concomitant symptoms, comorbidities, treatments, and prognosis.

**Results:**

The number of inpatients from outside our region increased from 9 in the second wave to 53 in the third wave. The initial manifestations were cold-like and gastroenteritis-like symptoms, gustatory and olfactory dysfunction being frequently occurring concomitant symptoms. On admission 32 patients had mild disease, 108 moderate I, 54 moderate II, and 6 severe. We divided the 200 patients into second and third wave groups and compared their baseline characteristics. The third wave group was older and had more severe disease. The main treatments implemented were dexamethasone and remdesivir. Three patients (1.5%) required ventilation and 12 (6.0%) died in hospital.

**Conclusions:**

We investigated 200 patients hospitalized for COVID-19 over a period of 6 months. The patients in the second wave were relatively young and most had mild disease. In contrast, the patients in the third wave were older and had more severe disease and higher in-hospital mortality.

## Introduction

COVID-19, which originated in Wuhan, China, is caused by severe acute respiratory syndrome coronavirus 2 (SARS-CoV-2) and is continuing to spread rapidly worldwide.^1^ It quickly spread across Japan after the outbreak in the cruise ship Diamond Princes, which occurred in the first week of February, 2020.^2^

On 18 February 2020, prior to the formal opening of our hospital, the Japanese Government asked us to admit 128 individuals for isolation and continued observation. They comprised asymptomatic SARS-CoV-2-infected persons and cabinmates who had been in close contact but had tested negative on the ship.^3^

Shortly thereafter, the number of COVID-19 patients rapidly increased in Tokyo and Osaka, in response to which the Japanese Government declared a state of emergency on 7 April 2020 in accordance with the Law on Special Measures against Novel Influenza. The area in which this act applied was subsequently expanded to include the entire country. The state of emergency was ended on May 25 in view of declining numbers of new cases of COVID-19.

We did not initially accept COVID-19 patients when our hospital opened on April 7. However, on July 22 we decided to prioritize admission of COVID-19 patients to our hospital in response to the second wave, which had begun in June.

Although the number of new patients decreased temporarily from September, it increased again from November, constituting the third wave. A second state of emergency was declared on 7 January 2021. This ended on 18 March 18 in response to a reduction in number of infections. During this period, many COVID-19 patients were admitted to our hospital.

Despite being a medium-sized regional hospital with 400 inpatient beds, we received requests for admission of COVID-19 patients both from within and outside our region.

Results of large-scale epidemiological investigations have been reported from various countries, including Japan; however, few have been from non-urban, regional healthcare settings.

Therefore, we here report on the status of patients with COVID-19 who had been admitted to and discharged from our hospital over a six-month period spanning the second and third waves of infection in Japan.

## Methods

The study cohort comprised 200 COVID-19 patients who had been admitted to our hospital from 22 July 2020 to 31 January 2021.

The primary indications for hospitalization were moderate disease II (requiring oxygen supplementation) and severe disease (Table 1, Reference 4: COVID-19 treatment guidelines in Japan).^4^ Additionally, some older patients, patients with comorbidities, including immunologically compromised patients, and pregnant women were also referred for hospitalization from the prefecture or public health centers as medically indicated.

The primary discharge criteria included 10 days from symptom onset and resolution of symptoms for 72 hours.^4^ Those with pneumonia were assessed by a physician to determine whether they could safely be discharged home or back to a long-term care facility.

## Results

**Figure 1:**
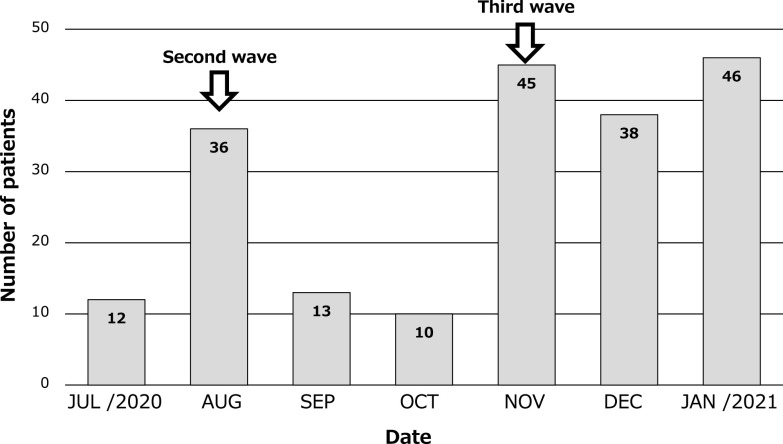
Monthly number of patients hospitalized for COVID-19. In July, there were 12 patients, but in August there were 36, which was the second wave. The number decreased to 13 in September and 10 patients in October, but increased rapidly to 45 patients in November, marking the third wave. In December there were 38 patients and in January there were 46 patients.

The numbers of patients with COVID-19 hospitalized per month from 22 July 2020 to 31 January 2021 are shown in Figure 1. The total number was 200. In July, there were 12 patients, but in August there were 36, this coinciding with the second wave. There were only 13 new patients in September and 10 in October. Thus, 71 patients were hospitalized for COVID-19 during the second wave. In November, the number increased rapidly to 45 patients, this coinciding with the third wave. In December 38 patients were hospitalized, as were 46 in January. Thus, a total of 129 patients were hospitalized for COVID-19 during the third wave.

**Figure 2:**
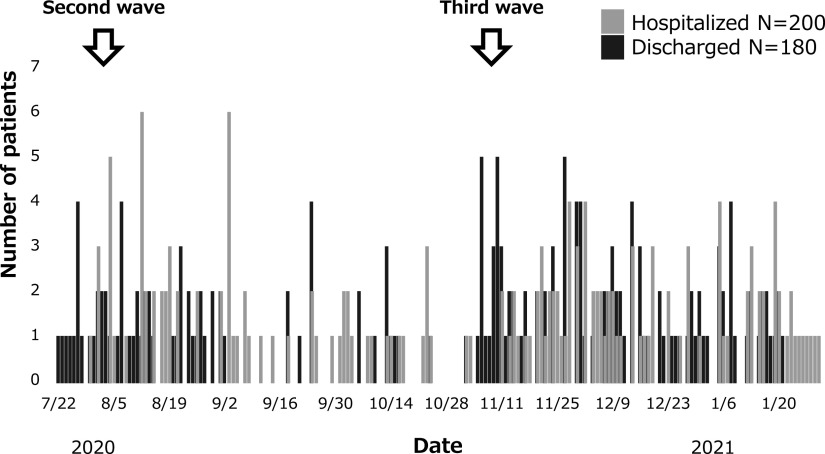
Daily number of patients with COVID-19 hospitalized and discharged. Silver bar is number of hospitalized patients, black bar is number of discharged patients.

The numbers of patients with COVID-19 hospitalized and discharged daily from 22 July 2020 to 31 January 2021 are shown in Figure 2.

**Figure 3:**
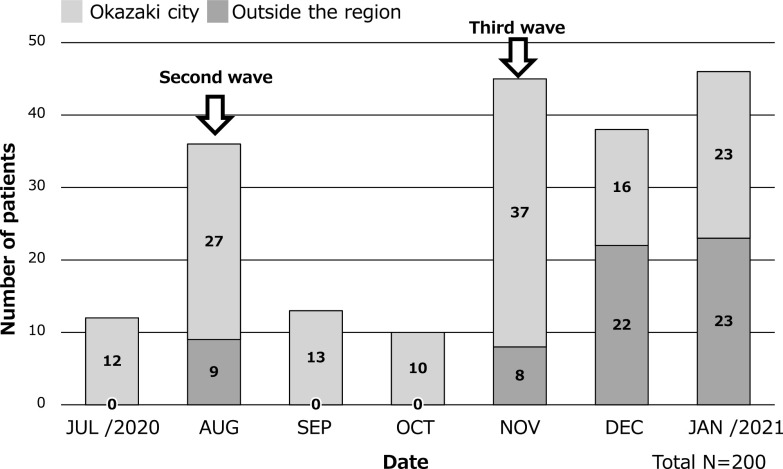
Number of COVID-19 inpatients from Okazaki city or outside the region. In the second wave, only 9 patients from outside the region were hospitalized in August, but in the third wave, the number of inpatients increased to 8 patients in November, 22 patients in December, and 23 patients in January. Silver bar is number of hospitalized patients from Okazaki city, dark brown bar is number of hospitalized patients from outside the region.

The number of patients hospitalized for COVID-19 from outside the region from 22 July 2020 to 31 January 2021 are shown in Figure 3. During the second wave, only nine patients from outside the region were hospitalized (in August). However, during the third wave, 8 such patients were admitted in November, 22 in December, and 23 in January, making a total of 53 patients, most of whom were older individuals from long-term care facilities.

The characteristics of the 200 patients are shown in Table 2. Their average age was 61.3 years (range 17–104 years); 117 were men and 83 women. The main presumed sources of transmission were long-term care facilities in 47 patients, family members in 39, restaurants in 25, night clubs in 19, hospitals in 10, and unknown in 49. The main comorbidities were hypertension in 59 patients, diabetes mellitus in 35, dementia in 24, hyperlipidemia in 17, and respiratory disease in 15. The main initial symptoms were fever in 65 patients, fatigue in 23, cough in 22, and sore throat in 17, the main concomitant symptoms being gustatory dysfunction in 40 patients, fever in 32, cough in 26, and olfactory dysfunction in 22.

On admission 32 patients had mild, 108 moderate I, 54 moderate II, and 6 severe disease.

We divided the 200 patients into second and third wave groups. Patient characteristics according to these groups are shown in Table 3. The third wave group was older and had worse outcomes. Three patients (1.5%) required ventilators and 12 (6.0%) died in hospital.

The preadmission course of the patients is shown in Table 4. The interval between initial symptoms and polymerase chain reaction (PCR) testing averaged 4.5 days (range 3–18), the interval between PCR testing to hospitalization averaged 2.9 days (range 1–9), and the interval between initial symptoms and hospitalization averaged 7.4 days (range 1–20). The average length of hospitalization was 13.5 days (range 4–54).

The drugs administered to the patients are shown in Table 5. Dexamethasone was administered to 71 patients (to 36 as a single agent and to 35 in combination with other drugs). Remdesivir was administered to 30 patients (all in combination with dexamethasone). Anticoagulants (e.g., heparin, edoxaban) were administered to three patients (all in combination with dexamethasone). Ciclesonide inhalant was used in two patients (combined with dexamethasone). None of the patients received favipiravir, nafamostat mesylate, tocilizumab, or baricitinib.

## Discussion

In this study, the patients were relatively young and most of the ones in the second wave had mild disease. In contrast, the patients in the third wave were older and had more severe disease, with correspondingly more in-hospital deaths.

The COVID-19 Registry Japan (COVIDREGI-JP), a large-scale study in Japan, reported that the second wave was less severe than the first.^5^ In the third wave, the duration of hospitalization was longer because the patients were older and had more severe disease. As a result, the number of inpatients per day gradually increased. Mechanical ventilation was required by three patients.

COVIDREGI-JP has reported that patients aged over 60 years have more severe disease. However, patients aged over 80 years less frequently request aggressive treatment than do patients in their 60s and 70s. As a result, critical care interventions such as ECMO (extracorporeal membrane oxygenation) are infrequently implemented in patients aged over 80.^2^

In the present study, many patients had comorbidities, the main ones being hypertension, diabetes mellitus, dementia, hyperlipidemia, and respiratory disease. Studies in China, the USA, and the UK have reported that cardiovascular disease, diabetes mellitus, COPD and obesity are the most common comorbidities in patients with severe disease.^1,6,7,8^

The most common initial manifestations were cold-like and gastroenteritis-like symptoms. Many patients had concomitant symptoms, mainly gustatory dysfunction, fever, cough, and olfactory dysfunction (only one person).

Although the initial symptoms were consistent with those reported from other countries, the concomitant symptoms of gustatory and olfactory dysfunction occurred frequently. This apparent discrepancy may be attributable to media reports highlighting gustatory and olfactory disfunction as unique symptoms of COVID-19 at the end of March 2020, likely increasing awareness of these symptoms among the public and thus leading to these symptoms being reported more frequently than in other studies.^2^ However, in the present study, few patients reported gustatory or olfactory dysfunction as an initial symptom.

The average interval between onset and PCR testing was 4.5 days, indicating a slight delay in undergoing PCR testing. Nevertheless, the average interval between PCR testing and hospitalization was 2.9 days, indicating that patients were promptly referred for admission once the PCR test results were available.

The main drugs administered were dexamethasone,^9^ remdesivir,^10^ and anticoagulants.^4,11,12^ At that time, tocilizumab was not available for this purpose.^13^

As mentioned earlier, one reason for our low mortality rate is that, in Japan, hospitalization is often recommended even for mild cases. Accordingly, the characteristics of inpatients differ slightly from those of inpatients in other countries. Additionally, a possible explanation for the relatively low use of intensive care resources is that older patients tend not to request aggressive treatment.

The increase in the number of hospitalizations in the third wave was driven by referrals from outside the region. This likely reflected the marked increase in the number of infected patients from the second (nine) to the third (53) wave. Another factor influencing the number of admissions for COVID-19 in the third wave was that we accepted many patients with mild disease who had initially been confined to isolation hotels for observation and whose condition had subsequently deteriorated.

At the time of this writing, Japan is transitioning from the fourth to the fifth wave, the course of which is expected to be complicated by the emergence of highly transmissible variants that differ in type and rate of mutation between geographic regions. We hope that the data presented herein describing our experience in a community-based hospital in a non-urban setting will be helpful to providers in similar circumstances who are preparing for the next surge in COVID-19 cases requiring hospitalization.

### Study limitations

Although this was a single facility study, our hospital is registered for participation in the COVID-19 Registry Japan (COVIDREGI-JP), which is a large-scale study conducted in Japan. Therefore, the results of this study are included in the COVIDREGI-JP report. It is also necessary to increase the number of cases by continuing joint research with other institutions.

## Conclusions

We have here presented the clinical characteristics and courses of COVID-19 200 patients who were admitted to a community-based hospital in central Japan between July 2020 and January 2021. The second wave consisted of relatively young patients with mild disease, whereas older patients with more severe disease predominated in the third wave. The presenting symptoms were similar to those reported by others and dexamethasone and remdesivir were the most common treatments given. The higher case fatality rate in the third wave likely reflects the change in patient characteristics. This will likely change again in future waves when the majority of older individuals will have been fully vaccinated.

## Figures and Tables

**Table1:** Classification of COVID-19 severity

Severity	SpO_2_	Clinical status
Mild	SpO_2_≥96%	No respiratory symptom or only cough
Moderate I	93%<SpO_2_<96%	Dyspnea and pneumonia
Moderate II	SpO_2_≤93%	Requiring oxygen supplementation
Severe		Requiring ICU management or ventilator

**Table2:** Background of COVID-19 patients

N=200	
Age	Mean 61.3 years (17–104 years)
Sex	Males 117, Females 83
Sources of transmission	unknown 49, Long-term care facilities 47, family 39, restaurant 25, night clubs 19, hospital 10, workplace 5, church 5, karaoke 1, camp 1
Epidemiology	cluster 151, Non-cluster 49
Comorbidities	hypertension 59, diabetes mellitus 35, dementia 24, hyperlipidemia 17, respiratory disease 15, heart disease 15, cerebral infarction 9, hyperuricemia 7, psychiatric disorder 6, thyroid disease 5, cancer 3,cerebral hemorrhage 3, taking steroids 2 (multiple answers)
Initial symptoms	fever 65, fatigue 23, cough 22, sore throat 17, headache 8, anorexia 7, diarrhea 6, chills 5, joint pain 4, runny nose 3, vomiting 2, olfactory dysfunction 2, dysgeusia 1, sore throat 2, myalgia 1, dyspnea 1, nasal obstruction 1 (multiple answers)
Concomitant symptoms	dysgeusia 40, fever 32, cough 26, olfactory dysfunction 22, joint pain 7, headache 6, dyspnea 10, chest pain 5, sore throat 6, anorexia 9, myalgia 3, fatigue 6, lumbago 2, diarrhea 2, nasal obstruction 1, vomiting 3, dizziness 2, nausea 1, eruption 2, sputum 11, consciousness 3 (multiple answers)
Severity	mild 32, moderate I 108, moderate II 54, severe 6

**Table3:** Background of Second wave group and Third wave group

	Second wave group	Third wave group
N (M/F)	71 (47/34)	129 (70/59)
Age (mean)	50.8 years	66.8 years
Sources of transmission	Long-term care facilities 11	Long-term care facilities 36
Severity	mild 14, moderate I 44, moderate II 12, severe 1	mild 18, moderate I 64, moderate II 42, severe 5
Death discharge	1	11
Used Ventilator	0	3

**Table4:** Preadmission course of COVID-19 patients

Preadmission course	
1. The number of days from initial symptoms to PCR test	Mean 4.5 days (1–19 days)
2. The number of days from PCR test to hospitalization	Mean 2.9days (1–17 days)
3. The number of days from PCR test to hospitalization	Mean 7.4 days (1–19 days)
4. The length of hospitalization	Mean 13.5 days (2–54 days)

**Table5:** Drug treatments of COVID-19 patien

Drugs	
Favipiravir	0
Ciclesonide inhalant	2 (all in combination with dexamethasone)
Dexamethasone	71 (36 patients as a single agent, 35 patients in combination with other drugs)
Remdesivir	30 (all in combination with dexamethasone)
Anticoagulants (heparin, edoxaban, etc.)	3 (all in combination with dexamethasone)
Nafamostat Mesylate	0
Tocilizumab	0
Baricitinib	0
